# Development of Experimental Materials on Moral Judgment in Sport: Evidence From Chinese Athletes

**DOI:** 10.3389/fpsyg.2019.02802

**Published:** 2019-12-23

**Authors:** Zuosong Chen, Dong Wang

**Affiliations:** Department of Physical Education, Shanghai Jiao Tong University, Shanghai, China

**Keywords:** sport, misconduct, moral dilemmas, moral judgment, experimental material

## Abstract

**Objectives:** The existing scales for moral judgment in sport have some limitations when used for cognitive neural research. Developing a set of experimental materials with good validity is thus warranted. The purpose of this study was to develop experimental materials that can be used in cognitive neuroscience research on moral judgment in sport.

**Design:** Study 1 was a qualitative study and Study 2 used a within-subject design.

**Method:** In Study 1, a qualitative method was adopted to assess types of moral misconduct among Chinese athletes, based on news reports of Chinese athletes' moral misconduct collected from the Internet and from interviews with Chinese elite athletes. In Study 2, typical examples were selected from a qualitative analysis based on the types of moral misconduct observed among athletes in Study 1. The examples were then compiled, controlled, and modified. The validity of the developed experimental materials was evaluated.

**Results:** The moral misconduct observed in Chinese athletes can be divided into the following four categories: violent behavior, doping, match-fixing or tanking, and self-reported dishonesty. Subject analysis and item analysis consistently found that the experimental materials developed for moral judgment based on the four categories were significantly different in six aspects, including the rate of participants' agreement to the proposed resolution [*F*_Subject(3, 184)_ = 236.60, *p* = 0.00; *F*_Item(3, 156)_ = 471.17, *p* = 0.00], decision time [*F*_Subject(3, 184)_ = 23.69, *p* = 0.00; *F*_Item(3, 156)_ = 3.13, *p* = 0.03], moral conflict [*F*_Subject(3, 184)_ = 3.70, *p* = 0.01; *F*_Item(3, 156)_ = 10.71, *p* = 0.00], moral acceptability of the behavior [*F*_Subject(3, 184)_ = 58.22, *p* = 0.00; *F*_Item(3, 156)_ = 110.69, *p* = 0.00], emotional valence [*F*_Subject(3, 184)_ = 3.41, *p* = 0.02; *F*_Item(3, 156)_ = 3.11, *p* = 0.03], and emotional arousal [*F*_Subject(3, 184)_ = 1.32, *p* = 0.27; *F*_Item(3, 156)_ = 5.09, *p* = 0.00]. The experimental materials developed were not affected by the type of sport.

**Conclusions:** The developed experimental materials can be used as experimental materials for cognitive neuroscience research on moral judgment in sport.

## Introduction

Moral judgment in sport refers to the thought process that occurs when an athlete uses existing moral norms or standards to perceive moral phenomena in sport. It is a complex form of psychological processing (Wang and Chen, 2018). Currently, the scales used to measure moral judgment in sport can be divided into those which assess the stages of development of moral judgment (Bredemeier and Shields, 1984, 1986; Lind, 2006; Proios and Doganis, 2006; Mouratidou et al., 2007, 2008), those which assess value judgment in the sporting context (Hahm, 1989; Calmeiro et al., 2015), those which measure moral behavior judgment in sport (Gibbons et al., 1995; Stephens et al., 1997; Guivernau and Duda, 2002; Kavussanu et al., 2002; Sage et al., 2006; Lee et al., 2007; Romand et al., 2009; Malete et al., 2013; Whitehead et al., 2013; Gurpinar, 2014), and those which assess moral content judgment in sport (Proios, 2010). These scales have made important contributions to the study on moral judgment in sport. However, there are significant inadequacies in the direct use of these scales as experimental materials for cognitive neuroscience research on moral judgment in sport. Specifically, the research and development of the existing scales largely focused on revealing the stages of development of moral judgment in sport and on investigating the motivational mechanisms behind moral misconduct in sport, rather than assessing the behavioral choices that lead to such misconduct. In addition, the type of moral misconducts involved in these scales are limited by a lack of systematic and comprehensive organization and analysis of moral misconduct in the sporting context (Gibbons et al., 1995; Stephens et al., 1997; Guivernau and Duda, 2002; Kavussanu et al., 2002; Sage et al., 2006; Lee et al., 2007; Romand et al., 2009; Malete et al., 2013; Whitehead et al., 2013; Gurpinar, 2014). Meanwhile, moral dilemmas have become the most popular experimental paradigm in empirical studies on moral cognition, and have become a preferred paradigm in the field of cognitive neuroscience of moral judgment (Christensen and Gomila, 2012). Such a design is closer to real-life scenarios in sport, thereby improving the ecological validity of the related research. Moreover, existing scales primarily focus on a single sport (such as basketball or football) (Romand et al., 2009). Experimental materials regarding moral dilemmas that are applicable to a wide range of sports are still lacking, which leads to difficulties in conducting in-depth research on moral judgment in sport. Therefore, a systematic and comprehensive analysis of athletes' moral misconduct is needed. The development of experimental materials with moral dilemmas applicable to a wider range of sports is of great importance to further studies on moral judgment in athletes.

Furthermore, since the existing scales are mostly paper-and-pencil tests, items regarding moral dilemmas in sport fail to manipulate or control the many parameters involved in the dilemmas (such as type of dilemma, word count, style of expression, and sentence structure). There is also a lack of validity evaluation of the materials used (such as the emotional valence and arousal of materials regarding moral dilemmas in sport). Christensen and Gomila (2012) found that, when using moral dilemmas as the research paradigm, researchers tend to overlook the control and manipulation of the many variables involved in the moral dilemmas, which then significantly reduces the reliability of the research results. Christensen and Gomila (2012) systematically analyzed 25 experimental studies that used moral dilemmas as the research paradigm. They identified a total of 19 variables that required control and manipulation when adopting moral dilemmas in research. The 19 variables proposed by Christensen and Gomila (2012) served as a reference and basis for experimental studies on moral judgment based on the research paradigm of moral dilemmas.

In terms of the content, the existing scales on moral judgment in sport fail to conduct a systematic and comprehensive analysis of moral misconduct in sport. In terms of the presentation format, they fail to manipulate and control the many parameters that affect the research results. This will inevitably affect the reliability of the results of cognitive neuroscience research on moral judgment in sport to a certain extent. Therefore, in terms of cognitive neuroscience research on moral judgment in sport, the development of a set of experimental materials with good validity has become a crucial concern in need of an urgent solution. Two studies were conducted to develop a set of experimental materials which can be used in cognitive neuroscience research on moral judgment in sport. Study 1 aimed to reveal the types of moral misconduct in sport and collect examples of moral misconduct in sport through qualitative research. On the basis of Study 1, Study 2 compiled typical examples selected from Study 1 according to the types of moral misconduct in sport, and controlled and modified these based on Christensen and Gomila's (2012) suggestions, and assessed the validity of the experimental materials developed. The development of a set of experimental materials with good validity for cognitive neuroscience research on moral judgment in sport is an important and novel contribution of the present study. The present data was collected from a Chinese sample, though according to Hauser et al. (2010), moral judgments were found to be largely similar across ethnicity, religion, and nationality. Therefore, the experimental materials developed in this study might be extended to other cultural contexts. It is our hope that our study may have positive and practical effects on the cognitive neuroscience research on moral judgment in sport.

## Study 1: A Qualitative Study of Moral Misconducts Among Chinese Athletes

### Research Objective

Moral dilemmas have been a preferred paradigm in cognitive neuroscience research on moral judgment for decades, and these dilemmas often derive from classic philosophical dilemmas or adapted versions. Thus, the possibility of their happening in real life has been questioned (Wang, 2017). In order to enable athletes to better integrate into the context of moral dilemmas, Study 1 aimed to reveal the types of moral misconduct in Chinese athletes and collect examples of moral misconduct in sport through a qualitative analysis of online news reports and through interviews with high-performing Chinese athletes. This served to provide operational material for research on and the development of experimental materials on moral judgment in sport.

### Methods

#### Sources of Data

##### Source of Online Data

News reports of Chinese athletes' moral misconduct between 2000 and 2017 collected from authoritative websites including sports.sohu.com, sports.sina.com, sports.163.com, sports.qq.com, sports.xinhuanet.com and sportsonline.com.cn. The names of Olympic sports and some common non-Olympic sports, combined with search terms “暴力” (baoli, violence), “恶意犯规” (eyi fangui, intentional fouls), “兴奋剂” (xingfen ji, doping), “消极比赛” (xiaoji bisai, match-fixing or tanking), “失范行为” (shifan xingwei, misconducts), “弄虚作假” (nongxu zuojia, fraud), “垃圾话” (laji hua, trashtalk), “假赛” (jiasai, match-fixing), were used for the retrieval and collection of related online news. After removing duplicate reports of the same event, repeated articles reposted on different websites, and irrelevant data, 598 news articles were collected.

##### Source of Interview Data

In addition to the retrieval and collection of relevant news reports on the Internet, 90 elite athletes in China were interviewed regarding moral misconduct in sport. Among the interviewees, 41 were males (45.56%) and 49 were female (54.44%), 27 (30%) were engaged in provincial competitions, and 63 (70%) were engaged in national competitions. Their average age was 20.72 ± 1.92 years and the average amount of training received was 8.12 ± 1.90 years. The sports in which the interviewees engaged included basketball, football, track and field, taekwondo, judo, free fighting, tennis, table tennis, volleyball, badminton, etc. Face-to-face interviews were conducted, mainly focusing on two interview questions, namely, “Have you ever committed or observed moral misconduct during a competition?” and “Can you describe what happened (time, location and reason)?” The interviews were conducted in the evenings at the office of the coach or instructor. Each interview lasted approximately 40 min. To minimize the influence of social desirability bias, process control methods were adopted. For instance, prior to the interview, the interviewer emphasized the anonymous and confidential nature of the interview as well as the importance of the truthfulness and accuracy of the athletes' self-reports for the research results.

##### Research Procedure

The Nvivo 8.0 software (QSR International Pty Ltd, Victoria, Australia) was used to manage and analyze the data. The 598 news reports collected from the Internet and interview recordings of 90 athletes were organized and transcribed into Microsoft Word documents, which were then imported into the Nvivo 8.0 software for analysis. Next, referring to grounded theory methodology, a three-stage coding method (open coding, axial coding, and selective coding) was utilized to analyze and process the collected data.

### Results

Phrases related to moral misconduct in Chinese athletes were extracted during reading and reflecting on the collected data. After the corresponding open coding, 26 effective free nodes were developed (Table 1), including pushing (number of nodes: 60), elbowing (65), punching (18), kicking (49), pulling (58), tripping (49), bumping (39), wrestling/yanking (51), intentionally sticking out one's foot to trip an opposing player (19), hacking (67), shoving (55), strangling (3), biting (4), using profanity (157), quarreling (42), insulting and swearing (40), criticizing and accusing (70), disregarding or ignoring injured athletes (39), refusing to help (31), use of stimulants (250), strategic acts of throwing a match (35), deliberate time-wasting or stalling (82), match-fixing (42), fraud (20), not making one's best efforts to win a match (27), and self-reported dishonesty (139). The 26 free nodes were then compared, analyzed, and modified to form six axial codes, including physical violence (537), verbal violence (309), cold violence (70), doping (250), match-fixing or tanking (206), and self-reported dishonesty (139).

**Table 1:** Axially coded information.

**Axial coding (number of nodes)**	**Original coding (number of nodes)**
Physical violence (537)	Pushing (60), elbowing (65), punching (18), kicking (49), pulling (58), tripping (49), bumping (39), wrestling/yanking (51), intentionally sticking out one's foot to trip an opposing player (19), hacking (67), shoving (55), strangling (3), biting (4)
Verbal violence (309)	Using profanity (157), quarreling (42), insulting and swearing (40), criticizing and accusing (70)
Cold violence (70)	Disregarding or ignoring injured athletes (39), refusing to help (31)
Doping (250)	Use of stimulants (250)
Match-fixing or tanking (206)	Strategic act of throwing a match away (35), deliberate time-wasting or stalling (82), match-fixing (42), fraud (20), not using one's best efforts to win a match (27)
Self-reported dishonesty (139)	Self-reported dishonesty (139)

**Figure 1:**
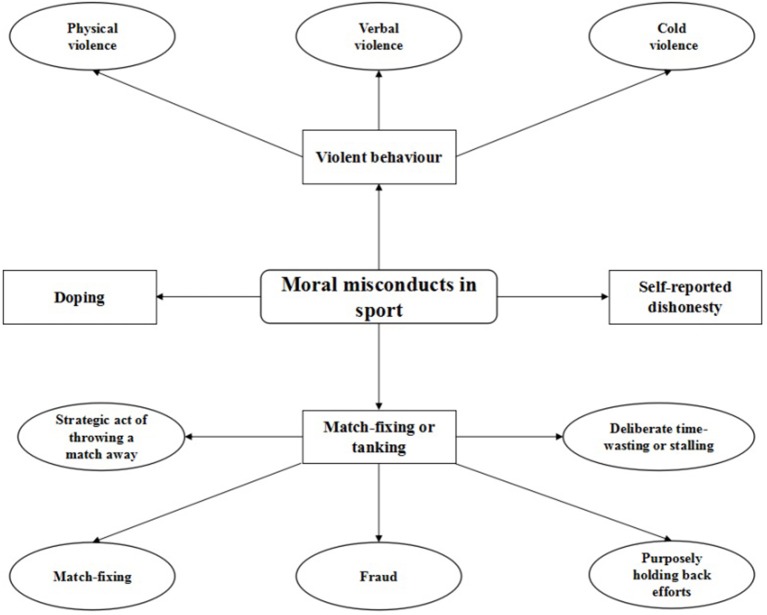
Types of moral misconducts among Chinese athletes.

Subsequently, four core categories emerged from the six axial codes (Figure 1). Among them, physical violence, verbal violence, and cold violence mainly reflected athletes' violent behavior in competitions and were categorized as violent behavior; use of stimulants was categorized as doping; behaviors such as playing games according to pre-determined results and not investing one's best efforts to win a match were categorized as match-fixing or tanking; lastly, self-reported dishonesty manifested misconduct that occurred during competitions but was only known to the athletes themselves and would not be discovered or detected by the referees and corresponding authorities. Self-reported dishonesty was a relatively special category as these cases were mainly collected from the interviews with the athletes and were seldom reported in the collected news reports. Unlike misconduct in the other three categories, self-reported dishonesty would not lead to negative consequences for the individuals exhibiting them. Through open, selective, and axial coding, the moral misconduct among Chinese athletes was grouped into the following four categories: violent behavior, doping, match-fixing or tanking, and self-reported dishonesty.

## Study 2: Development of Experimental Materials on Moral Judgment in Sport

### Research Objective

Study 2 aimed at developing experimental materials based on typical examples selected from Study 1, controlling and modifying the experimental materials, and assessing the validity of the experimental materials developed.

#### Participants

A total of 187 athletes were recruited from Shanghai, including 95 males (50.80%) and 92 females (49.20%). Among them, 156 (83.42%) participated in provincial competitions and 31 (16.58%) participated in national competitions. The average age of the participants was 20.84 ± 1.49 years, and the average amount of the training received was 9.04 ± 3.29 years. The sports in which they engaged included basketball, football, American football, fencing, taekwondo, track and field, wrestling, weightlifting, volleyball, badminton, swimming, boxing, gymnastics, judo, handball, and tennis. A small reward was given to participants after the completion of the experiment. The study was approved by the Ethics Committee of the Shanghai Jiao Tong University (no.: ML16026).

#### Material

The experimental materials stemmed from the typical examples obtained in Study 1. Based on the categories of the moral misconduct among Chinese athletes, the most common and influential examples were further selected for compilation (totaling 160 examples, 40 per category). The materials were compiled based on the following four principles: (1) the materials should involve moral conflict; (2) the wording and description should be colloquial and easy to understand; (3) the sentence patterns and structures should be consistent across different types of experimental materials; and (4) the athletes should be able to understand the experimental materials for all the sports involved. After compiling the preliminary version of the experimental materials, they were controlled and modified based on the 19 parameters proposed by Christensen and Gomila (2012). However, since the experimental materials in our study differed from those involving moral dilemmas in a real-life scenario, some of these parameters (such as speciesism, participants' demographic variables, kinship, intentionality, the trade-off, and certainty of event) were not suitable for the present study. Nevertheless, they were crucial to the construction of moral dilemmas in real-life situations. After deliberation and thorough consideration, presentation format, order of presentation, expression style, word count, participant perspective, type of question, and kind of moral transgression were selected as the parameters to control and modify the compiled experimental materials (Supplementary l). Finally, based on recommendations from previous studies (McGuire et al., 2009; Lotto et al., 2014), the present study assessed the validity of the experimental materials on moral judgment in sport based on six aspects, including the rate of participants' agreement to the proposed resolution, decision time, moral conflict, moral acceptability of the behavior, emotional valence, and emotional arousal, while conducting both subject and item analyses.

#### Experimental Design and Procedure

A within-subject design was adopted. The independent variables were the experimental materials on moral judgment regarding the four categories of misconduct. The dependent variables were the number of participants who agreed to the proposed resolution, decision time, ratings of moral conflict, moral acceptability of the behavior, emotional valence, and emotional arousal. The study was conducted in the Digital Language Laboratory at Shanghai University of Sport. Participants completed the experiment in small groups of 10 to 15 athletes each and were tested on computers in the same room. Specifically, before each trial, each participant was presented with one of the developed moral dilemmas on the screen. The participants could read the scenario at their own reading speed and then select “Enter” to proceed to the question-answer (QA) interface. The QA interface displayed five questions and options in the sequence presented in Table 2. Upon completion of the five questions, the computer program automatically displayed the next dilemma. The official experiment consisted of four blocks, each containing 40 moral dilemmas (10 per category). Furthermore, to minimize the influence of the social desirability bias, the researchers emphasized the confidentiality and anonymity of the answers as well as the importance of truthful and accurate answers to the research results before the official experiment commenced. Materials from the four categories in each block were also presented randomly. Moreover, a counter-indicative design was applied to the items regarding self-reported dishonesty. After completing each block, the computer program automatically displayed a message that suggested the participant take a break. The participants could decide on the duration of the rest time based on how tired they were. The entire experiment lasted around 40–50 min. All the data were automatically uploaded from the computer to the research supervisor's host computer for storage and backup.

**Table 2:** Question and answer sequence.

**Question**	**Answer sequence**
Q1. Would you do that?	The participants were required to choose between “Yes” and “No” (displayed on the same screen as the question, for all questions) based on the previously mentioned sport-related scenario. To balance the numbers of options presented on the left and right side, some participants were asked to press the letter “Z” on the keyboard for “Yes” and “M” for “No,” whereas others were asked to do the opposite. The time that each participant spent on answering the questions was also recorded
Q2. Does the described scenario involve moral conflicts?	The participants responded to the items on a 5-point scale, with “1” denoting “Totally Disagree” and “5” denoting “Totally Agree.” They were required to press the corresponding numbers on the keyboard to select from the five options
Q3. Do you consider this behavior morally acceptable?	The participants responded to the items on a 5-point scale, with “1” denoting “Totally Disagree” and “5” denoting “Totally Agree.” They were required to press the corresponding numbers on the keyboard to select from the five options
Q4. How pleasant do you feel after reading the scenario?	A set of pictures of a cartoon man were displayed below the question, with different facial expressions representing the changes in emotional valence, from “1” (“Very Unpleasant”) to “9” (“Very Pleasant”). Higher scores indicated a higher level of emotional valence experienced by the participants (Lang et al., 1993)
Q5. How emotionally aroused (nervous) are you after reading the scenario?	A set of pictures of a cartoon man were displayed below the question, with changes in the heart representing the changes in emotional arousal, from “1” (“Very Calm”) to “9” (“Very Nervous”). Higher scores indicated that the participants felt more nervous (Lang et al., 1993)

#### Data Processing and Analysis

In this study, E-Data Aid (Psychology Software Tools, Pittsburgh, PA) was employed to merge, screen, analyze, and pre-process the collected data. This study used the experimental materials in the four categories as the independent variable, and the number of participants who agreed to the proposed resolutions, decision time, moral conflict, moral acceptability, emotional valence, and emotional arousal as the dependent variables. SPSS 16.0 (IBM Corporation, New York, USA) was used for conducting descriptive statistical analysis and independent ANOVAs. Furthermore, each dependent variable was examined by both subject and item analyses. In the subject analysis, the dependent variable was the mean response of each participant to the experimental materials in each category. A one-way repeated measures ANOVA was adopted to analyze the dependent variable and when there is a serious violation of sphericity assumption, the multivariate tests would be adopted. In the item analysis, the dependent variable was the mean response of all participants to the experimental materials in each category. A one-way ANOVA was performed to analyze the data. The least significant difference (LSD) test was used for *post-hoc* comparison at subject analysis and item analysis. In addition, an ANOVA was conducted between sports (sports in which the participants engaged and sports involved in the experimental materials) and the dependent variables in order to explore the influence of the type of sport on the dependent variables.

### Results

#### Results of Formulating Experimental Materials on Moral Judgment in Sport

Based on the principles of compilation, the 160 typical examples collected were compiled into a preliminary draft. Next, the draft was controlled and modified based on the control variables proposed by Christensen and Gomila (2012), including participant perspective, presentation format, order of presentation, expression style, word count, type of question, and kind of transgression. After preliminary compilation, control, and modification, a total of 160 experimental materials (40 per category) were developed (Table 3).

**Table 3:** Examples of experimental materials on moral judgment in sport.

**Type of moral misconducts**	**Experimental materials on moral judgment in sport**
Violent behavior	I am participating in an important 1,500 meter final. I am in the lead up until the final stage but an opponent is fast approaching. The only way to prevent the opponent from surpassing me without the referee noticing is to play little tricks like pulling him/her. However, such behavior is against the rules
Doping	I am preparing for an important weightlifting final but I am significantly overweight for my weight class. I must find a way to reduce my weight as quickly as possible. One day I learned about a new diuretic drug that aids in rapid weight loss while being undetectable in drug tests. However, taking performance enhancing drugs is against the rules
Match-fixing or tanking	I am participating in an important badminton match. My teammate has already advanced to the semi-finals. If I win this game I will play against my teammate in the semi-finals. In order to avoid the encounter and maximize my chances of winning the medal I could choose to deliberately lose this game. However, such behavior is against sportsmanlike conduct
Self-reported dishonesty	I am in the middle of an important football final. The shot I kicked helped the team win the game. However, I knew that the ball actually went into the goal through the side net but the referee and other people didn't notice. I could choose to tell the referee the truth but it will cost our team the championship

*Table 3 only contains some examples of the experimental materials on moral judgment in sport. Please refer to the Supplementary 2 for the complete list and contents of the developed materials. The results of the ANOVA showed no significant differences in word count among materials from the four categories [F_(1, 156)_ = 0.82, p > 0.05]*.

#### Validity of Experimental Materials on Moral Judgment in Sport

**Figure 2:**
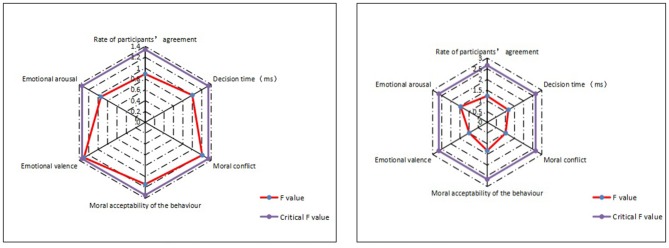
ANOVA results by sports based on the subject analysis and item analysis. Note: In the subject analysis (left), the critical *F* value was *F*_(60, 684)_ = 1.34, *p* = 0.05. The participants were engaged in 16 types of sports, among which various track and field events were all categorized as “track and field” during the analysis; In the item analysis (right) the critical *F*_(31, 128)_ = 1.54, *p* = 0.05. The developed materials on moral judgment involved 32 types of sports, among which various track and field events were all categorized as “track and field” during the analysis.

Subject analysis (Table 4) showed that the experimental materials developed for moral judgment based on the four categories were significantly different in six aspects (except for emotional arousal): the rate of participants' agreement to the proposed resolution [*F*_(3, 184)_ = 236.60, *p* = 0.00], decision time [*F*_(3, 184)_ = 23.69, *p* = 0.00], moral conflict [*F*_(3, 184)_ = 3.70, *p* = 0.01], moral acceptability of the behavior [*F*_(3, 184)_ = 58.22, *p* = 0.00], emotional valence [*F*_(3, 184)_ = 3.41, *p* = 0.02], and emotional arousal [*F*_(3, 184)_ = 1.32, *p* = 0.27]. Moreover, the sports in which the participants engaged showed no significant differences in six aspects (Figure 2): the rate of participants' agreement to the proposed resolution [*F*_(60, 684)_ = 0.89, *p* = 0.71], decision time [*F*_(60, 684)_ = 1.00, *p* = 0.49], moral conflict [*F*_(60, 684)_ = 1.21, *p* = 0.14], moral acceptability of the behavior [*F*_(60, 684)_ = 1.14, *p* = 0.22], emotional valence [*F*_(60, 684)_ = 1.30, *p* = 0.07], and emotional arousal [*F*_(60, 684)_ = 0.94, *p* = 0.61]. This indicated that the experimental materials developed were not affected by the types of sport in which the participants engaged.

**Table 4:** Results of the subject analysis.

	**Type of moral misconducts in sport**				**Variance analysis**	
	**VB**	**DP**	**MFT**	**SRD**	**Subject** **analysis**	**LSD**
**Variable**	***M ± SD***	***M ± SD***	***M ± SD***	***M ± SD***	***F*_**(3, 184)**_**	***p***
Participants' agreement (%)	27.63 ± 21.80	22.15 ± 33.42	39.85 ± 25.69	86.59 ± 17.68	236.60^**^	*p1* = 0.05; *p2* = 0.00; *p3* = 0.00; *p4* = 0.00; *p5* = 0.00; *p6* = 0.00
Decision time (ms)	1226.9 ± 496.55	1113.05 ± 591.20	1376.96 ± 634.09	1384.10 ± 662.37	23.69^**^	*p1* = 0.00; *p2* = 0.00; *p3* = 0.00; *p4* = 0.00; *p5* = 1.00; *p6* = 0.00
Moral conflict	4.13 ± 0.42	4.15 ± 0.46	4.10 ± 0.41	4.12 ± 0.46	3.70^*^	*p1* = 0.87; *p2* = 0.18; *p3* = 1.00; *p4* = 0.03; *p5* = 1.00; *p6* = 0.87
Moral acceptability of the behavior	2.62 ± 0.79	2.24 ± 0.94	2.80 ± 0.80	3.01 ± 0.81	58.22^**^	*p1* = 0.00; *p2* = 0.00; *p3* = 0.00; *p4* = 0.00; *p5* = 0.00; *p6* = 0.00
Emotional valence	4.60 ± 1.30	4.64 ± 1.42	4.64 ± 1.25	4.69 ± 1.28	3.41^*^	*p1* = 1.00; *p2* = 0.35; *p3* = 0.02; *p4* = 1.00; *p5* = 0.33; *p6* = 1.00
Emotional arousal	4.15 ± 1.55	4.08 ± 1.62	4.15 ± 1.54	4.16 ± 1.56	1.32	*p1* = 0.42; *p2* = 1.00; *p3* = 1.00; *p4* = 0.31; *p5* = 1.00; *p6* = 0.53

VB, violent behavior; DP, doping; MFT, Match-fixing or tanking; SRD, Self-reported dishonest;

** p < 0.01,

* *p < 0.05; p1: the significance value for the test of difference between violent behavior and doping; p2: the significance value for the test of difference between violent behavior and match-fixing or tanking; p3: the significance value for the test of difference between violent behavior and self-reported dishonesty; p4: the significance value for the test of difference between doping and match-fixing or tanking; p5: the significance value for the test of difference between match-fixing or tanking and self-reported dishonesty; and p6: the significance value for the test of difference between doping and self-reported dishonesty*.

Item analysis (Table 5) showed that the four categories were significantly different in six aspects: the rate of participants' agreement to the proposed resolution [*F*_(3, 156)_ = 471.17, *p* = 0.00], decision time [*F*_(3, 156)_ = 3.13, *p* = 0.03], moral conflict [*F*_(3, 156)_ = 10.71, *p* = 0.00], moral acceptability of the behavior [*F*_(3, 156)_ = 110.69, *p* = 0.00], emotional valence [*F*_(3, 156)_ = 3.11, *p* = 0.03], and emotional arousal [*F*_(3, 156)_ = 5.09, *p* = 0.00]. In addition, the sports involved in the experimental materials showed no significant differences in six aspects (Figure 2): the rate of participants' agreement to the proposed resolution [*F*_(31, 128)_ = 1.23, *p* = 0.21], decision time [*F*_(31, 128)_ = 1.16, *p* = 0.28], moral conflict [*F*_(31, 128)_ = 1.01, *p* = 0.46], moral acceptability of the behavior (*F* = 1.32, *p* = 0.14), emotional valence [*F*_(31, 128)_ = 0.99, *p* = 0.50], and emotional arousal [*F*_(31, 128)_ = 1.46, *p* = 0.07]. This also indicated that the experimental materials developed were not affected by the types of sport involved in the experimental materials.

**Table 5:** Results of the item analysis.

	**Types of moral misconducts in sport**				**Variance analysis**	
	**VB**	**DP**	**MFT**	**SRD**	**Item** **analysis**	**LSD**
**Variable**	***M ± SD***	***M ± SD***	***M ± SD***	***M ± SD***	***F*_**(3, 156)**_**	***p***
Participants' agreement (%)	27.63 ± 9.32	22.16 ± 4.35	39.85 ± 12.89	86.59 ± 4.43	471.17^**^	*p1* = 0.00; *p2* = 0.00; *p3* = 0.00; *p4* = 0.00; *p5* = 0.00; *p6* = 0.00
Decision time (ms)	1226.96 ± 338.52	1113.05 ± 417.95	1376.96 ± 425.72	1384.10 ± 628.24	3.13^*^	*p1* = 0.28; *p2* = 0.15; *p3* = 0.13; *p4* = 0.01; *p5* = 0.95; *p6* = 0.01
Moral conflict	4.13 ± 0.06	4.15 ± 0.04	4.10 ± 0.04	4.12 ± 0.04	10.71^**^	*p1* = 0.00; *p2* = 0.00; *p3* = 0.47; *p4* = 0.00; *p5* = 0.05; *p6* = 0.00
Moral acceptability of the behavior	2.62 ± 0.30	2.25 ± 0.10	2.80 ± 0.21	3.01 ± 0.34	110.69^**^	*p1* = 0.00; *p2* = 0.00; *p3* = 0.00; *p4* = 0.00; *p5* = 0.00; *p6* = 0.00
Emotional valence	4.60 ± 0.14	4.64 ± 0.09	4.64 ± 0.19	4.69 ± 0.14	3.11^*^	*p1* = 0.21; *p2* = 0.15; *p3* = 0.00; *p4* = 0.83; *p5* = 0.12; *p6* = 0.08
Emotional arousal	4.15 ± 0.08	4.08 ± 0.08	4.15 ± 0.12	4.16 ± 0.10	5.09^**^	*p1* = 0.00; *p2* = 0.91; *p3* = 0.71; *p4* = 0.02; *p5* = 0.79; *p6* = 0.00

VB, violent behavior; DP, doping; MFT, Match-fixing or tanking; SRD, Self-reported dishonest;

** p < 0.01,

* *p < 0.05; p1: the significance value for the test of difference between violent behavior and doping; p2: the significance value for the test of difference between violent behavior and match-fixing or tanking; p3: the significance value for the test of difference between violent behavior and self-reported dishonesty; p4: the significance value for the test of difference between doping and match-fixing or tanking; p5: the significance value for the test of difference between match-fixing or tanking and self-reported dishonesty; and p6: the significance value for the test of difference between doping and self-reported dishonesty*.

## Discussion

### Types of Moral Misconduct Among Chinese Athletes

To collect operational materials for the development of experimental materials on moral judgment in sport, we conducted a qualitative study on the types of moral misconduct among Chinese athletes. The results showed that the six axial codes obtained through the qualitative analysis could be grouped into four core categories, namely, violent behavior, doping, match-fixing or tanking, and self-reported dishonesty. Our empirical results provided support for previous studies (Xiong et al., 2007; Li et al., 2013; Wu and Cao, 2016). Specifically, in terms of violent behavior, physical violence is the main form of violent behavior among Chinese athletes. This could be attributed to the fact that competitive sports emphasize intense competition, which is likely to motivate physical violence among players in the same game and provoke conflicts among athletes, between athletes and referees, or among the spectators (Xiong et al., 2007). This disrupts the normal order of the game and leads to serious consequences (even injuries and casualties). Therefore, people tend to pay more attention to physical violence. Verbal violence was also an important manifestation of violent behavior among Chinese athletes, as the number of nodes was second only to physical violence. One likely explanation was that, compared to physical violence, the consequences of verbal violence tend to be less serious (Wang, 2009). In addition, there were fewer news reports of verbal violence than of physical violence, and the former was mentioned more frequently during the interviews with athletes. Cold violence was also a manifestation of violent behavior among Chinese athletes; the number of nodes was less than those of physical and verbal violence, possibly due to the less frequent occurrence of cold violence.

Doping was also a common form of moral misconduct among Chinese athletes, which is in accordance with previous studies (Wu and Cao, 2016). Doping refers to situations in which improper human interference is used to improve one's performance in competitions. It is not only against the Olympic spirit but also prone to cause a certain level of harm to athletes' health. However, incidences of doping still exist despite repeated prohibitions. Therefore, doping has become one of the main types of moral misconduct of Chinese athletes. In terms of match-fixing and tanking, match-fixing or tanking was to achieve a certain goal, such as obtaining a better ranking and personal interests by deliberately losing a game (Zhang and Bi, 2014). Although some studies pointed out that it was unclear whether match-fixing or tanking violates sports ethics or the Olympic spirit (from the strategic viewpoint, for example) (Xiong et al., 2007), the present study showed that, based on the objective and motivation behind such behavior, both match-fixing and tanking are indeed against the Olympic spirit; they ought, therefore, to be categorized as moral misconduct in sport.

Self-reported dishonesty mainly refers to misconduct that occurs during competitions but is only known to the athletes themselves. Due to the special nature of this category, the cases were mainly collected from the interviews with athletes and were less frequently discovered in news reports. This was possibly due to the distinctive characteristic of behavior in this category—self-consciousness. Unlike the other three categories, self-reported dishonesty would not be discovered by the referees or corresponding authorities. In other words, such behavior had no negative consequences for the athletes, while benefiting them and/or their team.

### Validity of the Experimental Materials on Moral Judgment in Sport

The validity of the experimental materials was evaluated from six aspects, including the rate of participants' agreement to the proposed resolution, decision time, moral conflict, moral acceptability, emotional valence, and emotional arousal. In addition, both subject and item analyses were conducted (McGuire et al., 2009). The present study showed that the lowest of participants' rate of agreement with the solution (utilitarian choice) was doping, and the highest was self-reported dishonesty. A possible explanation for such findings is that doping not only violates the Olympic spirit but also harms the athletes' health. In addition, the Chinese government has undertaken promotional and educational anti-doping initiatives (Wu and Cao, 2016). Doping also had the lowest score on moral acceptability, which indirectly explained the above findings. The rate of utilitarian choice was the highest in the scenarios with self-reported dishonesty due to the characteristic of this type of misconduct. Since the misconduct in this type of scenario was often only known to the athletes themselves, even though they chose to commit them, such misconduct would go unnoticed by the referees or authorities. Therefore, individuals tend to make utilitarian choices in judgment scenarios that are unbeknownst to anyone but them.

With regard to the decision time, the present results showed that the decision time regarding doping was the shortest, followed by violent behavior, while for scenarios with match-fixing or tanking and self-reported dishonesty it was longest. As mentioned earlier, doping violates the Olympic spirit and is harmful to athletes' physical health. Therefore, when faced with doping scenarios, fewer participants made a utilitarian choice, and consequently, the decision time was shorter. In the case of match-fixing or tanking and self-reported dishonesty, the athletes might have to weigh the pros and cons of each option repeatedly; hence, it took them longer to reach a decision. In terms of moral conflict, the present results revealed significant differences among the materials in different categories. In addition, all of the categories had relatively high scores for moral conflict, and there was no dilemma without any moral conflict. This indicated that the developed materials involved a satisfactory level of moral conflict. On the other hand, compared to the other categories, doping had the highest scores for moral conflict. Again, this finding could be attributed to the fact that it violates sports ethics and the Olympic spirit, and is harmful to athletes' health. Moreover, the moral acceptability of doping was also the lowest among all of the categories.

The scores for moral acceptability were significantly different across the four categories. Doping had the lowest score for moral acceptability, suggesting that it was generally disapproved of by most of the athletes. The scores for emotional valence and arousal were found to differ significantly across different categories (except for emotional arousal scores in the subject analysis). This indicated that all of the dilemmas were able to provoke feelings of disgust and nervousness among the participants. Since all the dilemmas involved moral conflicts, making a utilitarian judgment meant having to violate sports ethics or the Olympic spirit, while not making a utilitarian judgment meant having to lose the game or affect the chance of winning. Therefore, the participants would have to face negative consequences regardless of whether they chose to make a utilitarian choice. However, although all the moral dilemmas in the four categories triggered disgust and nervousness, such emotions were not as strong as the ones found by Lotto et al. (2014). This may be due to the fact that all the dilemma scenarios constructed in this study were common cases that frequently occur in competitive sports, while most of the real-life moral dilemmas compiled by Lotto et al. (2014) involved extreme life-or-death decisions in a philosophical context.

The results showed that the sport in which the participants engaged and those involved in the experimental materials had no influence on participants' agreement to the proposed resolutions, decision time, moral conflict, moral acceptability of the behavior, emotional valence, and emotional arousal. These findings showed that the experimental materials were not affected by the type of sport. Athletes of all sports could easily comprehend and respond to the developed materials from all the four categories. Therefore, these materials can be used as valid experimental materials for conducting cognitive neuroscience research on moral judgment in sport.

## Conclusions

The current study suggested that the moral misconduct among Chinese athletes could be categorized into violent behavior, doping, match-fixing or tanking and self-reported dishonesty. The experimental materials on moral judgment in sport were significantly different across the four categories in terms of the rate of participants' agreement to the proposed resolution, decision time, moral conflict, moral acceptability of the behavior, emotional valence, and emotional arousal. In addition, the results of the subject and item analyses were consistent. The experimental materials developed were not affected by the type of sport. The findings indicated that the developed experimental materials can be used as experimental materials for conducting cognitive neuroscience research on moral judgment in sport.

## Data Availability Statement

The datasets generated for this study are available on request to the corresponding author.

## Ethics Statement

The studies involving human participants were reviewed and approved by the Ethics Committee of the Shanghai Jiao Tong University (no. ML16026). The patients/participants provided their written informed consent to participate in this study.

## Author Contributions

DW and ZC: conceptualization, investigation, methodology, writing—original draft preparation, and writing—review and editing. ZC: supervision and funding acquisition. All authors read and approved the final version.

### Conflict of Interest

The authors declare that the research was conducted in the absence of any commercial or financial relationships that could be construed as a potential conflict of interest.
