# Supplementary material for: Development of Experimental Materials on Moral Judgment in Sport: Evidence From Chinese Athletes
Source: Front Psychol. 2019 Dec 23;10:2802. doi: 10.3389/fpsyg.2019.02802 (PMC6935579; doi:10.3389/fpsyg.2019.02802)
Supplement: Supplementary file 1 [file Table_1.docx]

**Supplementary 1 List of paramenters used for controlling and modifying the complied experimental meterials**

In terms of the presentation format, as the developed materials were mainly used to conduct cognitive neuroscience research on moral judgment in sport, textual images were displayed on the computer screen (in Songti font, black, and size 18, with a grey background). The order of presentation was standardized across the experimental materials in the four categories. In other words, the sentence structure used in the materials was roughly the same throughout. In terms of expression style, Christensen and Gomila (2012) believe that the expression style of moral dilemmas could affect participants’ decision-making. Specifically, rhetorical and emotional expressions are more likely to affect the experimental results because they may activate emotion-related brain areas during decision-making. Therefore, the expressions of materials in the four categories were unified and a flat and straightforward expression style was adopted, without words or phrases that convey rich emotions. With regard to the word count, Christensen and Gomila (2012) pointed out that there were apparent differences in the average word count between the impersonal and personal materials developed by Greene et al. (2001), which could affect the research results. In particular, they suggested that the results of studies that apply event-related potential (ERP) and functional magnetic resonance imaging (fMRI) techniques could be affected. Hence they suggested that, when constructing experimental materials regarding moral dilemmas, researchers should balance the word count across different types of dilemmas. Therefore, using the most concise expressions possible, our study balanced the numbers of words across the materials in the four categories. In terms of the selection of participant perspective, differences in the perspective assigned to the participants in the moral dilemma scenarios could also influence their moral judgment (Royzman & Baron, 2002). To help the participants immerse more fully in the scenarios, all the materials were presented from a first-person perspective. Studies have also found that differences in the type of questions affect participants’ moral judgment. For instance, O'Hara et al. (2010) found that people tended to consider moral vignettes with the word “wrong” as a more serious transgression than those with words such as “forbidden”. Hence, our study unified the type of questions across materials in the four categories with the yes–no question “Would you do it?” . In terms of participant perspective, to help the participants immerse more fully in the scenarios, all the materials were presented from a first-person perspective. According to Christensen and Gomila (2012), the kind of transgression, that is, whether the misconduct violates the Five Foundations of Morality, autonomy, or divinity, also affects one’s judgment during moral dilemmas. The moral transgressions involved in the experimental materials compiled in the present study manifested as the choice between personal or team interests and sports ethics or the Olympic spirit. Thus, there was no difference in the type of transgression among the materials in the four categories.

**References**

Christensen, J. F., & Gomila, A. (2012). Moral dilemmas in cognitive neuroscience of moral decision-making: A principled review. *Neuroscience and Biobehavioral Reviews, 36*(4), 1249-1264. doi:10.1016/j.neubiorev.2012.02.008

Greene, J. D., Sommerville, R. B., Nystrom, L. E., Darley, J. M., & Cohen, J. D. (2001). An fMRI investigation of emotional engagement in moral judgment. *Science, 293*(5537), 2105-2108. doi:10.1126/science.1062872

O'Hara, R. E., Sinnott-Armstrong, W., & Sinnott-Armstrong, N. A. (2010). Wording effects in moral judgments. *Judgment and Decision Making, 5*(7), 547-554. doi:10/101109/jdm101109

Royzman, E. B., & Baron, J. (2002). The preference for indirect harm. *Social Justice Research, 15*(2), 165-184. doi:10.1023/A:1019923923537
