# Supplementary material for: Development of Experimental Materials on Moral Judgment in Sport: Evidence From Chinese Athletes
Source: Front Psychol. 2019 Dec 23;10:2802. doi: 10.3389/fpsyg.2019.02802 (PMC6935579; doi:10.3389/fpsyg.2019.02802)
Supplement: Supplementary file 2 [file Table_2.docx]

**Supplementary 2 The developed experimental materials on moral judgment in sport**

**Violent Behavior**

1. I am participating in an important basketball final, but in recent years our team has consistently been runner-up due to the fierce competition. When the game is in overtime, I need to flagrant foul the opposing super player and force his/her leave the field. This will help my team win even though that might hurt his/her.

2. I am taking part in an important sailing final. In the final stage of the game, I see the first boat next to me capsize due to improper operation. As a result the athlete fell into the water. I could stop to help his/her, but this would affect my success.

1. I am taking part in an important basketball final. We were initially in the lead but eventually overtaken by the opposing team after several controversial calls made by the referee. The team became very upset believing the decisions were unfair. As the captain I felt like I needed to do something to teach the referee a lesson but it could hurt the referee.
2. I am playing in an important football final. When the game is about to end, the score was tied. It was clear that the opposing player had a clear shot to make a goal. I was close to the player but because of the poor defensive positioning I could only attack from behind which would injure the player.
3. I am participating in an important speed skating final against an opposing player who keeps blocking my progress. There is only one more lap to skate around in the game. The only way to prevent the player from winning without the referee noticing is to play little tricks like pulling the athlete's clothes. However, such behavior is against the rules.
4. I am playing in an important football final, the opposing player is aggressive and play little tricks to elbow me which angers me. I could attack this player with the ball by tacking for revenge but this could hurt his/her.
5. I am participating in an important 800 meters track final, but the opposing player has been using his/her body to block my progress. At this point, there is only one last corner left in the race. The only way to overtaking him/her without the referee noticing is to do something sneaky like pushing his/her. However, such behavior is against the rules.
6. I am playing in an important football final. The game is about to end and I am in the penalty area where the opponent's defense is strong. The only way to score a goal to help the team win without the referee noticing is to pushing them. However, such behavior is against the rules.
7. I am participating in an important water polo final. My responsibility is to mark the opposing striker, but with little affect. The game has entered the final stage and the only way to win the game without the referee noticing is to play little tricks like “black feet” to make him/her leave the field. However, such behavior is against the rules.
8. I am participating in an important football final, but it’s difficult since the other team has a skillful offensive super-shooter. At the beginning of the game, I can deliberately step on the player to make it impossible for him/her to continue participating in the game. This will help the team win the game but it might hurt him/her.
9. I am taking part in an important cycling race. I am in the leading position up until the final stage but an opponent is close on my tail. The only way to prevent him/her from surpassing me without the referee noticing is to suddenly cut in front of his path. However, this might be dangerous for the opponent.
10. I am playing in an important football final. At this point, the game is about to end and the score is tied. I break through the defensive line while at this time the opponent's goalkeeper has also fallen to the ground. My focus is on trying to score a goal to help the team win the game but this will hurt the goalkeeper.
11. I am participating in an important basketball final, but the opposing player frequently interferes with my play which makes me angry. I can seek revenge by elbowing the player without the referee noticing but this will cause the player to be injured.
12. I am running an important marathon. A kilometer from the finish line, I find that the top runner fell to the ground due to muscle cramps. I can stop to help the player, but it will cost my title.
13. I am playing in an important football final. The game is about to end and the score is tied, the opposing player broke through my defense with the ball. The only way to prevent this player from scoring is to deliberately kick the player but it will hurt him/her.
14. I am participating in an important Chinese freestyle martial arts competition. The competitor and I are equally skilled so the game is extremely intense. The game has entered the final stage and the only way to win the game without the referee noticing is to play little tricks. But if I do this it will hurt the opponent.
15. I am playing in an important basketball final. Whenever I get the ball an opponent frequently insults me with derogatory and inappropriate word which makes me upset. When the opponent has the ball I can retaliate against him/her in the same way. However, such behavior is against sportsmanlike conduct.
16. I am playing in an important marathon swimming competition. I am in the leading position up until the final stage when an opponent catches up to me. The only way to prevent the opponent from surpassing me is to use my body to ram him/her. However, such behavior will hurt him/her.
17. I am participating in an important football final and the teammate’s mistakes has allowed the other team to tie with us. At this point, the game is about to end but the teammate makes another mistake causing the team to lose which makes me very angry. I could blame him/her, but such behavior is against sportsmanlike conduct.
18. I am participating in an important basketball final and my team is likely to win since I’ve been playing well. Then a fan of the opposing team throws a drink at me which makes me very angry. I could rush to beat up the fan, but such behavior is against sportsmanlike conduct.
19. I am playing in an important rugby final. The game is about to end and the score is tied. My teammate successfully takes the ball towards the end zone but the opposing player quickly returns to the defense. The only way I can hinder him/her defense is to tackle this player. However, such behavior will hurt him/her.
20. I am playing in an important hockey final. The game is about to end and we are in the lead by 1 point. The opposing player launched a last minute attack to break through my defense. The only way to prevent this player from scoring is to hook him/her with my stick. However, such behavior will hurt him/her.
21. I am playing in an important football final. The referee misjudged my actions when I was fighting with the opponent. I knew I didn't foul but the referee adhered to his/her original decisions even after I argued with the referee. I can berate the referee, but such behavior is against sportsmanlike conduct.
22. I am participating in an important basketball final, but I was accused by the fans because of the poor performance on the field. At this point, I just got on the game, the fans frequently abused me, which made me very angry. I can fight back the fans in the same way, but such behavior is against sportsmanlike conduct.
23. I was participating in an important gymnastics final but failed to win the championship because of a mistake. My teammate harassed me for failing which made me feel embarrassed and upset. In the follow-up competition, my teammate lost the championship because of the same mistake. I could also make fun of him/her. However, such behavior is against sportsmanlike conduct.
24. I am taking part in an important football final. The game has entered the final stage and my team is one point behind. The opponent’s teammate faked a fall to deliberately instigate a fight with me in effort to delay the game. I could accuse the opponent and play into the fight but such behavior is against sportsmanlike conduct.
25. My teammate and I are competing in an important speed walking race. At one kilometer away from the finish line my teammate who was in the lead suddenly fainted to the ground. I could stop to help him/her but this will hinder my success.
26. I am participating in an important football tournament. I was fighting with my opponent for the ball and I knew getting it was important for our victory. The only way to prevent the opponent from getting the ball is to make some illegal moves like elbowing. However, this will hurt him/her.
27. I am participating in an important basketball final. Due to the malicious foul made by the opposing player, my teammate got injured and forced to sit out of the rest of the game. The team was very angry. In order to avenge our teammate I could foul the opposing player in the same way but this will hurt him/her.
28. I am participating in an important football match. The game is about to end and the score is tied. I managed to intercept a play on our side and make a break to the opponent’s side. As I approached the goalie I found that the opponent's goalkeeper suddenly fainted and no one else saw that because everyone was still at the other end of the field. I could stop from shooting and call for help, but this will affect our team's victory.

31. I am participating in an important 1500 meter final. I am in the lead up until the final stage but an opponent is fast approaching. The only way to prevent the opponent from surpassing me without the referee noticing is to play little tricks like pulling him/her. However, such behavior is against the rules.

1. I am playing in an important basketball final. The match was about to end and the score is tied. I prepared to launch the last attack but the ball was intercepted by the opponent player. The only way to prevent the player from launching fast attack is to pull on him/her. However, such behavior will hurt the player.
2. I am participating in an important boxing final. I learned that my opponent’s left shoulder was injured prior to today’s game and has not yet fully recovered. During the fight my tactic could be to frequently use heavy punches targeted on the injured shoulder. This will directly help me win the game but will aggravate the opponent's injury.
3. I am participating in an important basketball final. The game is coming to an end and our team is leading by 1 point. The [opposite](C:/Users/wangdong/AppData/Local/youdao/dict/Application/7.5.0.0/resultui/dict/?keyword=opposite) striker is preparing [driving](C:/Users/WangDong/AppData/Local/youdao/dict/Application/8.4.0.0/resultui/html/index.html#/javascript:;) [layup](C:/Users/WangDong/AppData/Local/youdao/dict/Application/8.4.0.0/resultui/html/index.html#/javascript:;). I am the closest defender and the only way to prevent the striker from scoring is to trip him/her. However, such behavior will hurt the player.
4. I am participating in an important marathon. I am in the lead and about to be overtaken by another racer. At this time both of us arrive at the last water refill station. I could spill the opponent's water bottle to interfere him/her and creating favorable conditions for win. However, such behavior is against sportsmanlike conduct.
5. I am participating in an important football final. The ball flies threw the air and whoever can get control of the ball will have an advantageous position. An opponent and I both anticipate the ball the head it. The only way to obtain the ball without the referee noticing is to play little tricks. However, such behavior will hurt the other player.
6. My partner and I are participating in an important badminton doubles competition. The game has reached the match point but we ended up losing because of a stupid mistake made by my partner. I could berate him/her for venting frustration, but such behavior is against the spirit of competition.
7. I am participating in an important hurdles final. I am in the leading position with just ten meters from the finish line. An opponent is gaining on me and about to gain my position. The only way to prevent the opponent from surpassing me without the referee noticing is to pulling him/her. However, such behavior is against the rules.
8. I am taking part in an important car race. In the final stage of the game I’m in second place. As I round a corner I see that the first player’s car slid and crashed in a mud pit. I can stop and help the player pull the broken car out, but this would affect my champion.
9. My teammates and I are competing in an important short-track speed skating final. My teammate is in the lead but about to be overtaken by an opponent. The only way that I can help my teammate win the championship is to push the opponent down. However, such behavior will hurt the opponent.

**Doping**

1. I am preparing for an important 1500m final but recently I've been performing poorly so I must find a way to solve this problem as fast as possible. One day, I find out about a new drug [hydrochlorothiazide](C:/Users/wangdong/AppData/Local/youdao/dict/Application/7.5.0.0/resultui/dict/?keyword=hydrochlorothiazide) that can help improve rapid sport performance and is undetectable in drug tests. However, taking performance enhancing drugs is against the rules.
2. I am preparing for an important 100 meter final but I have not been systematic training for long so I’m concerned about my performance. One day, I find out about a new drug testosterone that can help improve my athletic performance quickly and it’s undetectable in drug tests. However, taking performance enhancing drugs is against the rules.
3. I am preparing for an important discus final but I have not been training as long as other competitors. I’m concerned that my level will be lower than others. One day, I find out about a new drug GHRP that can help me to improve sport performance quickly and is undetectable in drug tests. However, taking performance enhancing drugs is against the rules.
4. I am preparing for an important 1500 meter swimming final. I have injuries that still impede my progress and growth. One day, I learned that a new drug [trimetazidine](C:/Users/wangdong/AppData/Local/youdao/dict/Application/7.5.0.0/resultui/dict/javascript:;) can help me improve athletic performance quickly and it’s undetectable in drug tests. However, taking performance enhancing drugs is against the rules.
5. I am participating in an important marathon. I'm incredibly exhausted due to poor endurance. At the water refill site, the coach told me that a new drug testosterone can help me recover my body quickly and it’s undetectable in drug tests. However, taking performance enhancing drugs is against the rules.
6. I am preparing for an important 200m swimming final. My endurance is very poor so I must find a way to solve this problem as fast as possible. One day, I find out about a new drug nandrolone that can help enhance my body's endurance rapidly and it’s undetectable in drug tests. However, taking performance enhancing drugs is against the rules.

7.I am preparing for an important judo final, but my body is exhausted due to over-training so I must find a way to solve this problem as fast as possible. One day, I learned that a new drug methyl hexylamine tablets can help me to eliminate fatigue quickly and it’s undetectable in drug tests. However, taking performance enhancing drugs is against the rules.

1. I am preparing for an important 100m swimming final but I am still overweight because I can’t control myself around food so must find a way to solve this problem as fast as possible. One day, I find out about a new drug Clenbuterol that can help me lose weight rapidly and it’s undetectable in drug tests. However, taking performance enhancing drugs is against the rules.
2. I am preparing for an important 100m final. I still haven’t fully recovered from an injury so I want to find a way to solve this problem as fast as possible. One day, I learned that a new drug oxilofrine can enhance my athletic performance quickly and it’s undetectable in drug tests. However, taking performance enhancing drugs is against the rules.
3. I am preparing for an important football draft. Recently I've been playing poor and must find a way to solve this problem as fast as possible. One day, I find out about a new drug ephedrine which can help me enhance my performance in time for the competition and it’s undetectable in drug tests. However, taking performance enhancing drugs is against the rules.
4. I am preparing for an important 50m rowing final. Even with training my upper body strength is still not strong enough so I want to find a way to solve this problem as fast as possible. One day, I learned that a new drug [clenbuterol](C:/Users/wangdong/AppData/Local/youdao/dict/Application/7.5.0.0/resultui/dict/?keyword=clenbuterol) can help me build muscle quickly and it’s undetectable in drug tests. However, taking performance enhancing drugs is against the rules.
5. I am preparing for an important 800m final. I have been getting professional training but not for a sufficiently long time so I must find a way to rapidly improve for the final. One day, I find out about a new drug nerobol that can help enhance sport performance and it’s undetectable in drug tests. However, taking performance enhancing drugs is against the rules.
6. I am running in an important marathon. I've been exhausted due to training in the hot weather so I want find a way to solve this fatigue as fast as possible. At the water refill site, the coach told me that a new drug tuaminoheptane can accelerate the body’s recovery and it’s undetectable in drug tests. However, taking performance enhancing drugs is against the rules.
7. I am participating in an important football final. I’m older than most players so I become exhausted easier. This effects my performance so I want to find a way to solve this problem as fast as possible. During a break I overhear players talking about a new drug clenbuterol that can help accelerate recovery and it’s undetectable in drug tests. However, taking performance enhancing drugs is against the rules.
8. I am preparing for an important 100 meter track race. I have not had professional training for a long time so I must find a way to improve my situation as fast as possible. One day I find out about a new drug oxilofrine that can help me to improve performance quickly and it’s undetectable in drug tests. However, taking performance enhancing drugs is against the rules.
9. I am preparing for an important fencing draft. I've been playing poorly which is not sufficient for the [competitive state](C:/Users/wangdong/AppData/Local/youdao/dict/Application/7.5.0.0/resultui/dict/javascript:;) level so I must find a way to solve this problem as fast as possible. One day I heard about a new drug nikethamide that can help me to enhance excitability that is undetectable in drug tests. However, taking performance enhancing drugs is against the rules.
10. I am preparing for an important rowing final. My muscle strength and tone are still insufficient for the race so I must find a way to solve this problem as fast as possible. One day I learned that a new drug norandrosterone can help accelerate muscle growth and it’s undetectable in drug tests. However, taking performance enhancing drugs is against the rules.
11. I am preparing for an important shooting tryout. Recently I’ve been feeling stressed out which has negatively affected my performance so I must find a way to solve this problem. One day I found out about a new drug [propranolol](C:/Users/wangdong/AppData/Local/youdao/dict/Application/7.5.0.0/resultui/dict/?keyword=propranolol) which stabilize the mind and it’s undetectable in drug tests. However, taking performance enhancing drugs is against the rules.
12. I am preparing for an important boxing final but I’m still too overweight for my weight group so I need to reduce my weight as fast as possible. One day I found out about a new drug nandrolone that accelerates weight loss and it’s undetectable in drug tests. However, taking performance enhancing drugs is against the rules.
13. I am participating in an important rugby final. During training I’ve been overexerting my strength so my body getting fatigued and sore which is limiting my progress. During pause of game, I overheard that a new drug methyltestosterone that accelerate the boy’s recovery time and it’s undetectable in drug tests. However, taking performance enhancing drugs is against the rules.
14. I am preparing for an important mountain bike race. I still haven’t fully recovered from previous injuries so I’m desperate to solve this problem as fast as possible. One day I learned that a new drug nandrolone decanoate that can help me recover and gain stamina quickly and it’s undetectable in drug tests. However, taking performance enhancing drugs is against the rules.
15. I was preparing for an important fighting competition. I am too overweight to enter the competition so I must find a way to reduce my weight as fast as possible. One day I found out about a new drug furosemide that can aid in rapid weight loss and is also undetectable in drug tests. However, taking performance enhancing drugs is against the rules.
16. I am preparing for an important 200 meter track final. I have not yet recovered from my injury and must find a way to solve this problem as fast as possible. One day I learned that the drug THG could be used to help rapidly improve my athletic performance while being undetectable in drug tests. However, taking performance enhancing drugs is against the rules.
17. I am playing in an important badminton final but I get too tired to continue playing the games. I must find a way to solve this problem as fast as possible. During pause of game, I overhear that the drug dexamethasone can enhances stamina quickly and is undetectable in drug tests. However, taking performance enhancing drugs is against the rules.
18. I am preparing for an important 100 meter hurdles final. I'm older than most players and physical fitness is still insufficient. I need to find a way to fix this as fast as possible. One day I learned that a new drug nandrolone can help improve athletic performance while being undetectable in drug tests. However, taking performance enhancing drugs is against the rules.
19. I am preparing for an important triathlon but I got injured in the early stages of my training. This has impeded on my progress so I must find a way to solve this problem so I can still participate in the race. Recently I learned that a new drug EPO can accelerate recovery while being undetectable in drug tests. However, taking performance enhancing drugs is against the rules.

27.I am preparing for an important 400m final. I’m always one of the last runners and my endurance is still lacking so I want to find a way to solve this problem as fast as possible. One day, I found out about a new drug [stanozolol](C:/Users/wangdong/AppData/Local/youdao/dict/Application/7.5.0.0/resultui/dict/?keyword=Stanozolol) that helps enhance endurance rapidly and goes undetected in drug tests. However, taking performance enhancing drugs is against the rules.

1. I am preparing for an important 5000 meters final. But due to my recent poor performance I may not be able to participate in the final. I must find a way to solve this problem as fast as possible. One day, I heard about the new peptide hormone that helps rapidly improve athletic performance and also goes undetected in drug tests. However, taking performance enhancing drugs is against the rules.
2. I am preparing for an important 100 meters final. I’ve had multiple injuries that still impedes on my progress so I must find a way to solve this problem as fast as possible. One day I found out about a new drug that rapidly improves athletic performance while being undetectable in drug tests. However, taking performance enhancing drugs is against the rules.
3. I am preparing for an important pole vaulting final. I got injured during training and must find a solution as fast as possible. One day I heard about a new drug codeine that can help relieve pain, improve athletic performance, and is undetectable in drug tests. However, taking performance enhancing drugs is against the rules.

31.I am preparing for an important weightlifting final but I am significantly overweight for my weight class. I must find a way to reduce my weight as quickly as possible. One day I learned about a new diuretic drug that aids in rapid weight loss while being undetectable in drug tests. However, taking performance enhancing drugs is against the rules.

1. I am preparing for an important bodybuilding championship but my muscle mass is insufficient compared to competitor standards. I must find a way to solve this problem as fast as possible so I can still participate. One day I learned about a new drug boldenone can helps rapid muscle gain and it’s undetectable in drug tests. However, taking performance enhancing drugs is against the rules.
2. I am preparing for an important swimming final. I am taking daily supplements that contain stimulants but I know they are not allowed to be taken for the race. One day I learned about a new drug [hydrochlorothiazide](C:/Users/wangdong/AppData/Local/youdao/dict/Application/7.5.0.0/resultui/dict/?keyword=hydrochlorothiazide) which can conceal stimulant ingredients so they go undetectable in drug tests. However, taking performance enhancing drugs is against the rules.
3. I am preparing for an important equestrian final but my horse got injured in a previous race so we might not be able to participate in the final. I soon learn that there is not going to be a drug test so there is an option to inject my horse with capsaicin to relieve his pain to ensure the championship. However, taking performance enhancing drugs is against the rules and cruel for the animal.
4. I am preparing for an important archery final. Recently my mood has been very volatile which has impeded my practice. One day I heard about a new drug [inderal](C:/Users/wangdong/AppData/Local/youdao/dict/Application/7.5.0.0/resultui/dict/?keyword=inderal) which is a mood stabilizer and it’s undetectable in drug tests. However, taking performance enhancing drugs is against the rules.
5. I am preparing for an important canoeing final but my physical training was delayed due to an injury. I’m desperate to find ways to improve my situation for the final. One day I heard about a new drug norandrosterone which rapidly gains and enhances stamina while going undetected in drug tests. However, taking performance enhancing drugs is against the rules.
6. I am preparing for an important road cycling race. I don’t my physical fitness is sufficient enough to take on this race so I must find a solution as fast as possible. One day I heard that a new drug [hemopoietin](C:/Users/wangdong/AppData/Local/youdao/dict/Application/7.5.0.0/resultui/dict/?keyword=hemopoietin) helps enhance physical ability and it’s undetectable in drug tests. However, taking performance enhancing drugs is against the rules.
7. I am preparing for an important gymnastic final but I am overweight which is preventing me from performing at my fullest potential. One day I heard about a new diuretic drug that helps rapid weight loss and it’s undetectable in drug tests. However, taking performance enhancing drugs is against the rules.
8. I am preparing for an important swimming entrance exam. My level is still far from reaching the reach the standard so I want to find a solution as fast as possible. One day I learned that a new drug [DHEA](C:/Users/wangdong/AppData/Local/youdao/dict/Application/7.5.0.0/resultui/dict/javascript:;) can help improve athletic performance quickly and it’s undetectable in drug tests. However, taking performance enhancing drugs is against the rules.
9. I am preparing for an important basketball final but I'll probably miss the game due to a knee injury. I really don’t want to miss this game so I’m desperate to find a solution as fast as possible. One day I learned that a new drug can help accelerate the recovery process and it’s undetectable in drug tests. However, taking performance enhancing drugs is against the rules.

**Match-Fixing or Tanking**

1. I will be participating in the 100m and 1500m swimming finals both occurring on the same day. According to the rules, the score of the 1500m is much higher than 100m. In order to save my energy for the second race and get more points, I could deliberately a false start during the first race. However, such behavior is against sportsmanlike conduct.
2. I am playing for an important shooting tryout against my best friend. I still have to play against him/her even though I have been selected in advance. In order to help my friend be selected, I could deliberately perform poorly in this game. However, such behavior is against sportsmanlike conduct.
3. I am participating in an important badminton match. My teammate has already advanced to the semi-finals. If I win this game I will play against my teammate in the semi-finals. In order to avoid the encounter and maximize my chances of winning the medal, I could choose to deliberately lose this game. However, such behavior is against sportsmanlike conduct.
4. I am participating in an important basketball group tournament. Only the winning team gains five points and advances to the next round. As game comes to an end and the score is still tied. In order to make the game go in to over time, I could deliberately make all my free throws without score. However, such behavior is against sportsmanlike conduct.
5. I am participating in an important football final. I’m in a tough situation because someone is blackmailing me to rig the game. Due to my gambling addiction I owe this person a huge sum of money. In order to pay off the debt, I could to accidentally kick the ball into my own goal. However, such behavior is against sportsmanlike conduct.
6. I am participating in an important tennis final. I’m exhausted from practicing in the hot weather which has forced me to overexert my body. The game has entered a key determining point. In order to have time to recover before starting the next round I could pretend to have cramps and request a medical break. However, such behavior is against sportsmanlike conduct.
7. I am playing in an important group match where both sides can go to the next round if there’s a draw. As the game is about to end and the score is tied I am given a penalty kick. In order to achieve a win-win situation, I cloud deliberately miss the kick. However, such behavior is against the spirit of competition.
8. I am going to participate in an important 110 meter hurdles final but I got an injury so I may not be able to participate. If I don’t participate I will need to return compensation to advertisers who sponsored me. In order to avoid giving compensation, I could fake an injury during the game. However, such behavior is against sportsmanlike conduct.
9. I am participating in the first round of a tennis tournament when my wife goes into labor. In order to end the game as soon as possible to accompany my wife, I could deliberately lose the game. However, such behavior is against sportsmanlike conduct.
10. I’m a goalkeeper and I’ll be playing in an important football final. I need money to pay for my family’s medical bills and I was propositioned to rig the game for the money. In order to pay for the medicals bills, I could let the other team score on me. However, such behavior is against sportsmanlike conduct.
11. I am participating in an important rugby final. Two of my teammates were sent off the field because of a controversial penalty which vexed the team. In order to show our dissatisfaction, As captain I could instigate a protest to stop the game. However, such behavior is against sportsmanlike conduct.
12. I am playing in an important basketball final but five teammates were suspended due to the naturalization issue. This made the team extremely upset. In order to show protest, As captain I could choose to organize the team to commit malicious fouls. However, such behavior is against the spirit of competition.
13. I am participating in an important ping pong final. My opponent took the lead after I made several mistakes. The game has entered a defining point that will establish the winner and I think it’s hopeless to win. In order to end the game as soon as possible, I could deliberate make mistakes. However, such behavior is against sportsmanlike conduct.
14. I am participating in an important epee final. I am exhausted from going against [fierce](C:/Users/wangdong/AppData/Local/youdao/dict/Application/7.5.0.0/resultui/dict/?keyword=fierce)[competition](C:/Users/wangdong/AppData/Local/youdao/dict/Application/7.5.0.0/resultui/dict/?keyword=competition). The game has entered the final round. In order to win the championship, I could choose to intentionally delay the game in the lead position. However, such behavior is against the spirit of competition.
15. I am participating in an important football match. The team that loses this game can avoid the defending champion team in the follow-up game. As the game comes to an end the score is still tied. My position is goalkeeper so I could allow the other team score on me so that our team loses. However, such behavior is against sportsmanlike conduct.
16. I am participating in an important taekwondo final. I have been exhausted from the physically intense fight even though I’ve gained control and the top position. In order to avoid the opponent's gaining the dominant position, I could choose to deliberately stall the game until time runs out. However, such behavior is against the spirit of competition.
17. I am participating in an important football match. The team that loses the game can avoid the defending champion team in the follow-up game. As game is coming to an end the score is still tied. In order to lose the game, I could deliberately fake an accident of kicking the ball into our own goal. However, such behavior is against sportsmanlike conduct.
18. I will be participating in the 100 m and 200 m swimming finals on the same day. I don’ t have the stamina to do both so the 100 m is priority because the score is more important according to the rules. In order to conserve strength but still win the championship of 100 m, I can choose to “swim slowly” in the 200 m finals. However, such behavior is against sportsmanlike conduct.
19. I am signing up for an important football final. I am over age according to the new rules. During the registration of the competition and I learned that there is no bone age assessment in the game. In order to help the team win the game, I could choose to age-fixing. However, such behavior is against sportsmanlike conduct.
20. I am participating in an important football final. The game has entered the final stage and our team is in the lead but the opponent's counterattack is extremely fierce. In order to prevent being overtaken, I could pretend an injury that makes it look like the opposing team was at fault so we can consume the game time. However, such behavior is against sportsmanlike conduct.
21. I am taking part in an important football final. We were once in the lead but were beaten by the opposing team after several controversial decisions. The team was very dissatisfied. In order to express dissatisfaction with the referee, I could organize a team strike. However, such behavior is against the spirit of competition.
22. I am participating in an important boxing final. I have become exhausted by the physical intensity. There is only one final round left in the game. In order to win, I could choose to keep him/her in a hold to deliberately delay the game time. However, such behavior is against sportsmanlike conduct.
23. I am playing in an important football final. The game is coming to an end and the score is still tied. I have the ball and advance toward the goal but because the opponent's guard is defending well the shot is hopeless. In order to win the game, I could choose to diving so I can take a penalty kick. However, such behavior is against the spirit of competition.
24. I am participating in an important judo final. My opponent is from the PLA team. According to the rules of the game even if they win the provincial team I represent will still receive a gold medal. In order to achieve a win-win, I could deliberately lose the game. However, such behavior is against the spirit of competition.
25. I’m a goalkeeper and I am participating in an important football final. The game has entered the final stage my team is in the lead but the opponent is making successful counterattacks. In order to win the game, I could choose to stall the game by changing gloves and tying shoes until the time ends. However, such behavior is against sportsmanlike conduct.
26. I am participating in an important kayaking race. Whoever loses the game can avoid racing the champion in the semi-finals. In order to get a better ranking, I could choose to deliberately lose the race to avoid competing the tougher competitor in the next class. However, such behavior is against sportsmanlike conduct.
27. I am participating in an important basketball final. The game is about to end my team is leading by 1 point. The opponent prepares to do a layup shot. If he/she succeeds then we will lose the game. In order to win the game, I could make an offensive foul by [flopping](C:/Users/wangdong/AppData/Local/youdao/dict/Application/7.5.0.0/resultui/dict/javascript:;). However, such behavior is against sportsmanlike conduct.
28. I am the goalkeeper and I am participating in an important football match. Winning and losing teams have already been secured but the team we are fighting now must win to avoid dropping in rank. I have been offered a tempting reward if I let the other team win. In order to get the money, I could choose to let the other team score. However, such behavior is against sportsmanlike conduct.
29. I am playing in an important tennis match. The opponent is a novice and there is no doubt that he/she will be defeated. During the break someone offers me money if I lose the game and the money sounds tempting. In order to get the money, I could pretend to be injured and give up. However, such behavior is against sportsmanlike conduct.
30. My partner and I are about to participate in an important badminton doubles match. However, due to the regulation change if we win the match we must go against another teammate. In order to avoid this situation, my partner and I decide to intentionally lose the game. However, such behavior is against sportsmanlike conduct.
31. I am participating in an important basketball scrimmage. The first team that scores five points gets to advance to the next round. The game is about to end and the team is only ahead by two points. In order to get to the next round, I could deliberately throw the ball into our basket which will force the game into overtime. However, such behavior is against sportsmanlike conduct.
32. I'm a key player and our team is playing in an important football match. I am over age according to the rules and cannot play on the field. The match is fierce and the score is tied. In order to help the team win the game, I could choose to impersonate my teammate participate in the competition. However, such behavior is against sportsmanlike conduct.
33. I am participating in an important fencing semifinal. The opponent is my teammate who is much more advanced than me. In order to help my teammate conserve strength, I can choose to deliberately lose the game quickly. However, such behavior is against sportsmanlike conduct.
34. I am playing in an important badminton semifinal. The opponent is my teammate and the winner of this game will face the seeded player in the final. I also don’t have an ideal record against the seeded player. In order to maximize the team's chances of winning a gold medal, I can let my teammate to win. However, such behavior is against sportsmanlike conduct.
35. I am participating in an important basketball match. The team who loses the game can avoid going up against the defending champions in the semifinal. The game is coming to an end and we are leading by one point. In order to lose the game, I could accidentally shoot in our hoop. However, such behavior is against sportsmanlike conduct.
36. I am participating in an important cycling competition. I’m cycling behind everyone because of an inefficient start. In order to regain a position, I could deliberately crash in the first half of the course. However, such behavior is against sportsmanlike conduct.
37. I am participating in an important judo final. I have been exhausted by the physical intensity although my score is ahead of my opponent. In order to win the game, I could choose to deliberately delay the game time by utilizing the hide or fastening belt tactics. However, such behavior is against sportsmanlike conduct.
38. I am participating in an important volleyball match. The winning team must go up against the champion team in the follow-up game. The game has entered a point that requires a tiebreaker. In order to lose the game, I could deliberately make mistakes while serving the ball. However, such behavior is against sportsmanlike conduct.
39. I am participating in an important billiards final. I don’t really have a chance of winning because my opponent is highly skilled. In order to end this game as soon as possible, I could deliberately breaking the red ball and create a scoring opportunity for the opponent. However, such behavior is against sportsmanlike conduct.
40. I am participating in an important car race. I can't advance to the next round even if I win because I lack a strong score from the previous rounds. In the final lap I am in the leading position. In order to help my teammate advance to the championship, I can give my position to my teammate by slowing down. However, such behavior is against sportsmanlike conduct.

**Self-Reported Dishonesty**

1. I am participating in an important rugby final. The team eventually won the game with the help of my touchdown. However, I knew that I had stepped out of the sideline while sprinting and no one else noticed. I could choose to tell the referee the truth, but it will cost our team the championship.
2. I am participating in an important badminton final. The opponent's kill was judged to have touched the net so I won the game. However I’m sure that the opponent's ball did not touch the net since I was closest to the net when it happened. I could choose to explain this to the referee, but it will cost my win.
3. I am participating in an important football match. I was given a penalty kick by referee after the opponent committed a foul when we got in a fight. But I knew that I should have been given a free kick instead of penalty kick. I could choose to tell the referee the truth, but it will cost our team the championship.
4. I am participating in an important basketball final. I made two free throws to help the team lead by one point and win the game. But I knew that during the second penalty I was on the foul line but no one noticed. I could choose to explain this to the referee, but it will cost our team's victory.
5. I am participating in an important volleyball final. My volley attack at the last minute helped our team win. However the goal was invalid since I was offside but and no one noticed. I could choose to explain this to the referee, but it will cost our team the championship.
6. I am playing in an important javelin final. My teammate's final throw exceeded mine which knocked down my rank. I saw that my teammate's performance was invalid since the javelin head did not borough deep enough in the. I could choose to tell the referee the truth, but it will cost our friendship.
7. I am playing in an important basketball final. I fall while defending the opponent’s attack so the opponent was sentenced a foul. But I knew that the opponent did not foul I was just clumsy and tripped over my feet. I could choose to explain this to the referee, but my team would be upset and it could hurt our chance of winning.
8. I am participating in an important handball final. My offensive score helped the team win the championship. But I knew that I had made an offensive violation and the referee and players didn’t notice. I could choose to explain this to the referee, but it look bad on my team.
9. I am participating in an important football match. I’m given a penalty kick by referee since the opponent made a foul when I fight with him/her in the penalty area. But I knew that the opponent didn’t foul and the referee didn’t notice. I could choose to explain this to the referee, but this could make my team upset and cost us the chance to win.
10. I am participating in an important ping pong final. I won the game because of the opponent's serve was judged out of bounds. However, I knew that the opponent's serve was inside the line but no one saw. I could choose to explain this to the referee, but it will cost my victory.
11. I am participating in an important football final. The opponent’s offense was judged to be out of bounds by the referee. But I clearly saw that the ball was inside the bounds and the referee just didn’t see clearly. I could choose to explain this to the referee, but it could cost our victory.
12. I am participating in an important basketball final. I made a foul on the opponent's shot and the referee called two free throws. But I knew that he/her should have given three free throws because the foul happened outside the three-point line. I could choose to explain this to the referee, but it will upset my team and cost us the victory.
13. I am participating in an important tennis final. I won the game with on the final serve. But I know that the ball fell out of bounds but the referee and opponent did not see clearly. I could choose to explain this to the referee but it will cost my victory.
14. I am participating in an important basketball final. The referee mistakenly awarded the ball to me when I marked an opponent. But I knew that I had touched the ball out of bounds and the referee did not see clearly. I could choose to explain this to the referee, but it is not good for the team.
15. I am participating in an important 200 m final. I took the lead and passed the line to win the championship following the final sprint. But I knew that I had started before the whistle blew and the referee and the others did not see clearly. I could choose to explain this to the referee, but it will cost me my the championship.
16. I am participating in an important basketball final. I gave my teammate a serve and then he passed it to another teammate who dunked the ball which helped us to win the game. But I knew that I had stepped over the line when I was serving and the referee did not notice. I could choose to explain this to the referee, but it may cost the team the victory.
17. I am participating in an important football match. I kicked the ball out of bounds when I was fighting with an opponent. But I got the ball because of the referee did not see clearly. I could choose to explain this to the referee, but it would not be beneficial for the team.
18. I am participating in an important long jump final. I gained rank after my last jump. But I knew that I had stepped on the line when I was running but the referee did not see clearly. I could choose to explain this to the referee, but it will cost me my the championship.
19. I am participating in an important basketball final. My break away layup helped the team to win the game. However I knew that I had traveled on my layup but the referee and the others did not see clearly. I could choose to explain this to the referee, but it will cost our team the championship.
20. I am participating in an important 800 m dash. I was the first to hit the line after the final sprint. But I knew that I had fouled earlier and the referee and the others did not see clearly. I could choose to explain this to the referee, but it will cost my victory.
21. I am participating in an important basketball final. At this point our team leads by one point and the opponent missed their last shot. But I knew that I had fouled when I was defending and the referee did not see it clearly. I could choose to tell the referee the truth, but it will cost our team the championship.
22. I am participating in an important 110 m hurdle final. I won the championship after the final sprint. But I knew that I had pulled on the second player’s clothes when I passed him/her and the referee did not see it clearly. I could choose to tell the referee the truth, but it will cost my victory.
23. I am playing an important basketball game. Our team won the game after making a one point layup. But I knew that I had fouled from violating an interference ball but the referee did not see it clearly. I could choose to tell the referee the truth, but it will cost our team the championship.
24. I am playing an important basketball game. I made a shot in the three pointer zone which helped the team win the game. But I knew that I had committed a three-second violation but the referee did not see it clearly. I could choose to tell the referee the truth, but it will cost our team the championship.
25. I am participating in an important football final. The opposing player's volley hit the goalpost and the ball bounced into the goal. But the goalkeeper caught it and threw it out before the ball fell and the referee did not see it clearly. I could choose to tell the referee the truth, but it is not good for the team to win.
26. I am taking part in an important 100 m relay race. At this point, I was the first to hit the line after completely the handing over the baton. But I knew that I had fouled because of the transfer happened outside the allowed area. However, the referee did not see it happen clearly. I could choose to tell the referee the truth, but it will cost our team the championship.
27. I am participating in an important basketball final. At this point I score a three pointer to help the team lead by one point. But I knew that I had stepped on the three pointer line when I jumped but the referee didn’t notice it clearly. I could choose to tell the referee the truth, but it is not beneficial for the team.
28. I am participating in an important basketball final. Our team won the game by one point after the opponent's last shot was blocked by me. But I knew that I had blocked it violated of interference rule but the referee did not see it clearly. I could choose to explain this to the referee, but it will cost our team the championship.
29. I am participating in an important basketball final. I fouled while defending the opponent so the referee gave the opponent one inbounds pass. But I knew that our team had fouled five times so the other team should have two free throws. I could choose to tell the referee the truth, but it is not beneficial for the team.
30. I am participating in an important table tennis final. My final serve made me win the game but I knew that my serve scratched the net even though the referee and the opponent did not see it clearly. I could choose to explain this to the referee, but it will cost me my win.
31. I am participating in an important ping pong final. I won the game after the other party's serve was judged out of bounds. But I knew that the opponent's serve had hit my clothes but the opponent and referee did not see it clearly. I could choose to explain this to the referee, but it will cost me my win.
32. I am participating in an important volleyball final. Our team won the game after the opponent's serve was judged out of bounds. But I knew that the serve graze my finger and the referee did not see it clearly. I could explain this to the referee, but it will cost our team the championship.
33. I am participating in an important tennis final. I won the game after the opponent's serve was judged out of bounds. But I clearly saw that the ball fall on the boundary line but the referee and opponent did not see it clearly. I could choose to explain this to the referee, but it will cost me my win.

34.I am in the middle of an important football final. The kick I shot helped the team win the game. However I knew that the ball actually went into the goal through the side net but the referee and other people didn’t notice. I could choose to tell the referee the truth, but it will cost our team the championship.

1. I am participating in an important football final. I fell out of the penalty zone because an opponent pushed me so the referee awarded a penalty. But I knew that the opponent did not foul and the referee did not see it clearly. I could choose to explain this to the referee, but it is not beneficial for my team.
2. I am participating in an important football tournament. My score at the end of the game helped us win. But I knew that I had committed a handball foul and the referee and other people did not notice it clearly. I could choose to tell the referee the truth, but it will cost of our team to drop rank.
3. I am participating in an important basketball final. The opponent hit three points in the last minute. But I found that because the scorekeeper neglected to add two points on the scoreboard it allowed our team to win. I could choose to tell the referee the truth, but it is not beneficial for my team.
4. I am participating in an important shot-put final. I won the championship after the last throw. But I knew that I had stepped out of the throwing ring during my throw. The referee and others did not see it clearly. I could choose to explain this to the referee the truth, but it will cost my win.
5. I am participating in an important badminton final. I won the game after the opponent's serve was judged out of bounds. But I knew that the opponent's serve hit right on the sideline but the referee and opponent did not see it. I could choose to explain this to the referee the truth, but it will cost my win.
6. I am participating in an important ping pong final. My final serve made me win the game. But I knew that my serve was violated a boundary rule and the referee did not see clearly was not aware of it. I could choose to explain this to the referee the truth, but it will cost my win.
